# More than one in three proxies do not know their loved one’s current code status: An observational study in a Maryland ICU

**DOI:** 10.1371/journal.pone.0211531

**Published:** 2019-01-30

**Authors:** Alison E. Turnbull, Caroline M. Chessare, Rachel K. Coffin, Dale M. Needham

**Affiliations:** 1 Outcomes After Critical Illness and Surgery (OACIS) Group, Johns Hopkins University, Baltimore, Maryland, United States of America; 2 Division of Pulmonary and Critical Care Medicine, School of Medicine, Johns Hopkins University, Baltimore, Maryland, United States of America; 3 Department of Epidemiology, Bloomberg School of Public Health, Johns Hopkins University, Baltimore, Maryland, United States of America; 4 Medical Intensive Care Unit, Johns Hopkins Hospital, Baltimore, Maryland, United States of America; 5 Department of Physical Medicine and Rehabilitation, School of Medicine, Johns Hopkins University, Baltimore, Maryland, United States of America; University of Technology Sydney, AUSTRALIA

## Abstract

**Rationale:**

The majority of ICU patients lack decision-making capacity at some point during their ICU stay. However the extent to which proxy decision-makers are engaged in decisions about their patient’s care is challenging to quantify.

**Objectives:**

To assess 1)whether proxies know their patient’s actual code status as recorded in the electronic medical record (EMR), and 2)whether code status orders reflect ICU patient preferences as reported by proxy decision-makers.

**Methods:**

We enrolled proxy decision-makers for 96 days starting January 4, 2016. Proxies were asked about the patient’s goals of care, preferred code status, and actual code status. Responses were compared to code status orders in the EMR at the time of interview. Characteristics of patients and proxies who correctly vs incorrectly identified actual code status were compared, as were characteristics of proxies who reported a preferred code status that did vs did not match actual code status.

**Measurements and main results:**

Among 111 proxies, 42 (38%) were incorrect or unsure about the patient’s actual code status and those who were correct vs. incorrect or unsure were similar in age, race, and years of education (P>0.20 for all comparisons). Twenty-nine percent reported a preferred code status that did not match the patient’s code status in the EMR. Matching preferred and actual code status was not associated with a patient’s age, gender, income, admission diagnosis, or subsequent in-hospital mortality or with proxy age, gender, race, education level, or relation to the patient (P>0.20 for all comparisons).

**Conclusions:**

More than 1 in 3 proxies is incorrect or unsure about their patient’s actual code status and more than 1 in 4 proxies reported that a preferred code status that did not match orders in the EMR. Proxy age, race, gender and education level were not associated with correctly identifying code status or code status concordance.

## Introduction

In the United States, the number of intensive care unit (ICU) beds and annual cost of critical care services are steadily increasing.[1] Simultaneously, the use of mechanical ventilation during the last month of life[2] and the proportion of hospitalized patients with advanced dementia who are mechanically ventilated[3] have risen markedly. Ethicists, health-services researchers, and clinicians have worried these simultaneous trends put patients at risk of over-treatment[4] as a result of both cognitive biases (i.e. the cascade effect [5]) and system-level phenomena (e.g. clinical momentum [6]).

In response to these concerns, critical care professional societies endorse shared decision making with the goal of choosing treatments that are “medically appropriate and consistent with the patient’s values, goals, and preferences”[7]. Most patients lack decision-making capacity at some point during their ICU stay, [8,9] and only a third of American adults creates a legal document describing their preferences[10]. Therefore ICU clinicians rely heavily on proxy decision-makers to report the goals of their critically ill loved ones and to participate in deliberation about preference-sensitive treatments. However, it is unclear how well these ICU proxies understand fundamental decisions about their loved one’s care, including how the medical team should respond in the event of acute respiratory failure or cardiac arrest.

To help address this issue, we conducted in-person, structured interviews with all available healthcare proxies present in our adult medical intensive care unit (MICU). Our objectives were two-fold: 1) to assess whether proxies know their patient’s actual code status as recorded in the electronic medical record (EMR), and 2) to determine whether code status orders reflect ICU patient preferences as reported by proxy decision-makers.

## Methods

### Setting

This study was conducted in the MICU at Johns Hopkins Hospital (JHH) in Baltimore, Maryland. JHH is an university-affiliated, teaching hospital with 1,177 beds in an urban environment. The MICU has 24 beds, admitted 1,550 patients in 2017, and specializes in the care of high acuity patients with complex medical diagnoses. Participants were recruited as part of a Phase I study[11] of a brief activation intervention to prepare family members of ICU patients to be effective proxy decision-makers. All interviews with proxies were conducted in-person, and outside of the patient’s room. Participants provided oral consent and received a $10 gift card at the end of the interview. Johns Hopkins Medicine Institutional Review Board Committee X approved this study number: IRB00080137. The IRB approved oral consent because participation did not involve procedures normally requiring written consent outside of the research context.

### Recruitment

Between January, 2016 and May, 2016, we screened the MICU census 7 days per week to identify eligible families for this study. Families [12] were eligible once their patient had been in the MICU for >24 hours and the family remained eligible for 7 consecutive days thereafter or until patient death or ICU discharge. Family members were excluded if they were non-English speaking, <18 years old, or never physically visited the MICU. We preferentially enrolled a patient’s healthcare agent(s) or surrogate(s), as defined by Maryland State Law, and refer to both agents and surrogates using the term “proxy” as recommended [13]. In Maryland, healthcare agents are individuals appointed in advance directives and their authority is defined by the individual creating the advance directive. If a patient has not named an agent or the agent is not available and the patient lacks decisional capacity, other individuals are approached to act as surrogates in a pre-specified hierarchical order dictated by the state.[14] If a legal proxy was not able to visit the MICU, an adult family member who was physically present in the MICU and interacting with the clinical team as a patient representative was approached for this study. If the initial family member enrolled was not the patient’s legal healthcare proxy, we continued to attempt to recruit the patient’s legal proxy, and included both interviewed family members in analyses.[15]

### Study procedures

Participating proxies provided their demographic information and then, as part of the parent study, were read aloud a graphically illustrated booklet explaining the role of a healthcare proxy in the ICU. The booklet was written at a Flesch-Kincard reading grade level of 3.8, required 3.5 minutes to read aloud, and can be accessed via the study website at www.piperscience.org/proxy-activation. The booklet did not mention code status orders or explain CPR. After reading aloud, study investigators gave each participant a copy of the booklet and asked 1 open-ended question and 1 multiple-choice questions about their patient’s goal of care. Response options for the multiple-choice question were derived from previous research categorizing the goals of hospitalized patients [16–18]. Next, we asked 2 multiple-choice questions about their patient’s current use of life support and preferred code status avoiding medical terminology. (**S1 Fig**) After enrolling 11 proxies, an additional question was added to the interview to determine if proxies understood their patient’s actual code status at the time of the interview. Additional data were collected from the EMR regarding patient age, sex, race, zip code, location prior to hospitalization, ICU admission diagnosis, use of life support, and actual code status at the time of the proxy interview, as well as subsequent hospital vital status. Patients without any code status order in the EMR at the time of the interview were recorded and analyzed as being full code because these patients will be resuscitated if indicated.

### Analyses

Tables were used to display 1) proxy’s report of the patient’s current code status versus actual code status in the EMR at time of interview, 2) proxy’s report of the patient’s preferred code status vs. actual code status, with a “concordant code status” defined as agreement between preferred and actual status, and 3) proxy report of the patient’s preferred code status versus reported patient goal.

Characteristic of proxies who incorrectly identified their patient’s code status or reported that they did not know their patient’s code status were compared to proxies who correctly identified code status using the Wilcoxon-Mann-Whitney two-sample test, the Chi-square test, and the Fisher’s exact test (if cell counts <10), as appropriate. Statistical significance was defined as a two-sided P-value <0.05. Proxies and patients with concordant versus discordant code status (as defined above) were compared using the same statistical tests. In addition to reporting the P-value for these comparisons, we report the absolute effect size (Cohen’s d) as a measure of the magnitude of the difference to help with appropriate interpretation of the comparisons.[19,20] We conducted the following sensitivity analyses: 1) comparing concordant versus discordant proxies excluding proxies who were unsure of the patient’s code status, 2) comparing concordant versus discordant proxies excluding interviewed family members who were not legal healthcare proxies, and 3) comparing proxies who were incorrect or unsure of their patient’s actual code status versus proxies who correctly identified code status excluding interviewed family members who were not legal healthcare proxies. Statistical analyses were performed using the R programming language version 3.3.2 (Vienna, Austria).

## Results

**Fig 1:**
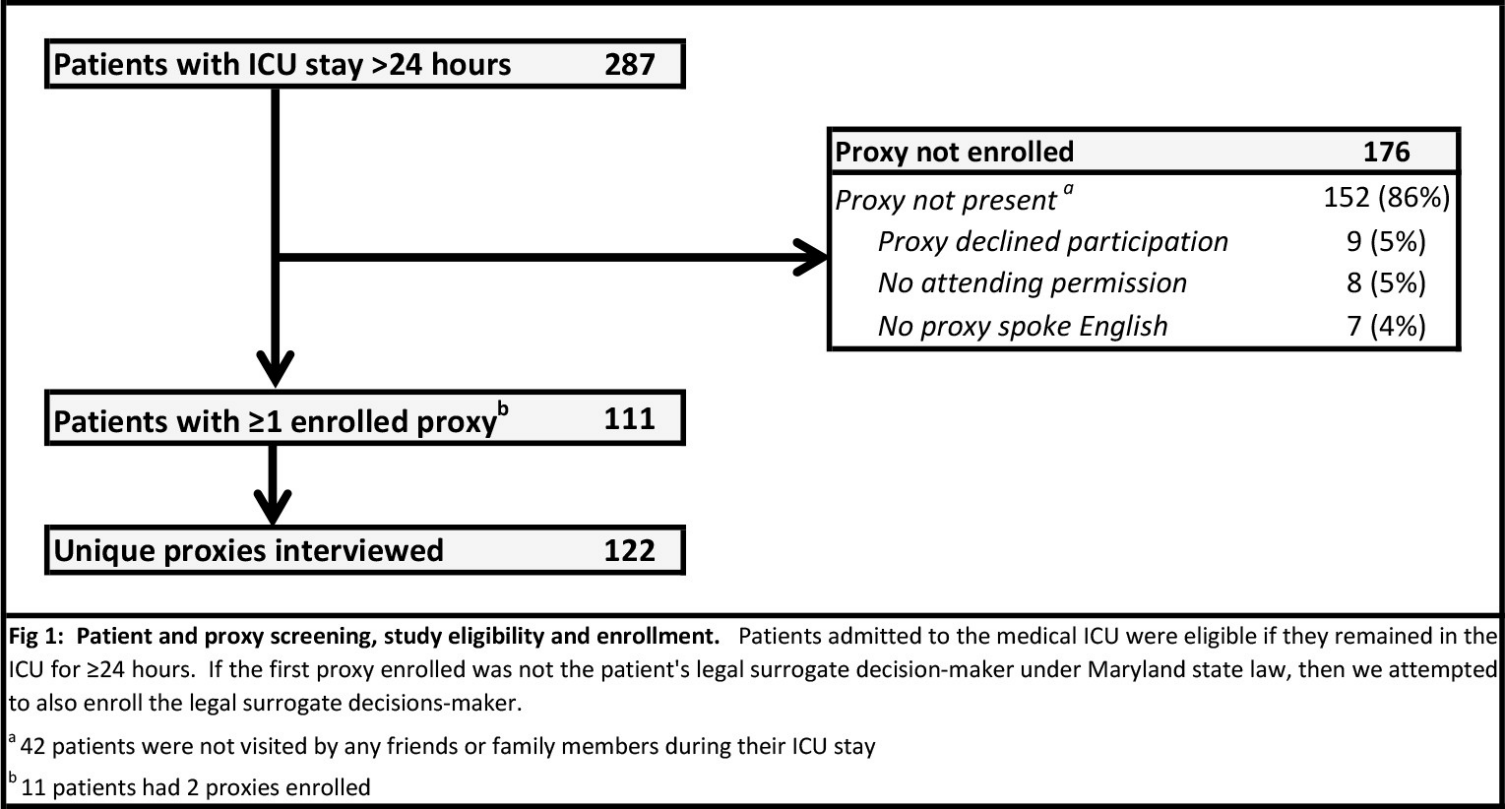
Patient and proxy screening, study eligibility and enrollment. Patients admitted to the medical ICU were eligible if they remained in the ICU for ≥24 hours. If the first proxy enrolled was not the patient's legal surrogate decision-maker under Maryland state law, then we attempted to also enroll the legal surrogate decisions-maker. ^**a**^ 42 patients were not visited by any friends or family members during their ICU stay ^**b**^ 11 patients had 2 proxies enrolled.

There were 122 unique family members interviewed for 111 consecutive eligible patients **(Fig 1).** Among these participants 79 (65%) were the patient’s legal healthcare proxy, median age was 51 years old (range: 18–79), 39 (30%) were male, 55 (45%) identified as Black or African-American, and median years of education was 14 (range: 10–24) **(Table 1)**. Five of the 111 patients (4%) had a legal document naming a healthcare agent in their medical chart.[15] Analyses regarding the external validity of this study sample have been previously reported.[15]

**Table 1:** Characteristics of 122 interviewed proxies.

Proxy Characteristics	N = 122
Age^a^ median (range)	51 (18–79)
Male^a^ n(%)	37 (30%)
Self-identified race^a^ n(%)	
Black or African American	55 (45%)
White	55 (45%)
Other	8 (7%)
Years of education, median (range)	14 (10, 24)
Have you ever supported a loved one in an ICU before?^a^ Yes(%)	75 (61%)
Patient's legal surrogate decision-maker	79 (65%)
Relation to patient^b^ n(%)	
Spouse/partner/girlfriend or boyfriend	46 (38%)
Adult child	40 (33%)
Parent	14 (11%)
Other family member	22 (18%)
Patient’s goal^a^^,^^c^ n(%)	
To be cured	31 (25%)
To live longer	22 (18%)
To improve health	38 (31%)
To maintain health	13 (11%)
To accomplish a personal life goal	2 (2%)
To be comfortable	13 (11%)
Unsure	2 (2%)
Patient’s preferred code status^d^ n(%)	
Use life-support machines to keep me alive no matter what. If my heart stops, do CPR.	48 (40%)
Use life-support machines to keep me alive no matter what. But if my heart stops don’t do CPR.	8 (7%)
Try to help me get better, but don’t use life support machines and if my heart stops don’t do CPR.	22 (18%)
Focus on keeping me as comfortable as possible, even if that means I die sooner.	11 (9%)
I don’t know what he/she would say.	31 (26%)

^a^ Proxies declined to report age (n = 2), sex (n = 2), race (n = 4), experience as an ICU proxy (n = 3), patient goal (n = 1), and patient’s preferred code status (n = 2).

^b^ Percentages do not add to 100% due to rounding.

^c^ Response to the question: *“In your opinion*, *which would of the following options best describes [patient’s name] goal right now*?*”*

^d^ Response to the question: *“In your opinion*, *how does [patient’s name] want doctors and nurses in the ICU to treat him/her*? *Which of the following statements sounds most like what [patient’s name] would say*?*”*

At the time of the 122 interviews with ICU proxies, 103 (84%) patients were full code. Among the 111 proxies asked to report their patient’s actual code status, 69 proxies (62%, 95% CI 52% -71%) were correct, 32 (29%, 95% CI 21% - 38%) were unsure, and 10 (9%, 95% CI 5% - 16%) were incorrect **(Table 2)**. Compared to the 69 proxies who correctly understood their patient’s code status, the 42 (38%) proxies who were incorrect or unsure had similar characteristics (P>0.20, effect sizes (*d*) ≤0.35) except for in admission diagnosis (P = 0.05, *d* = 0.98) **(S1 Table)**. Excluding interviewed family members who were not legal healthcare proxies did not qualitatively changed these findings (**S3 Table**).

**Table 2:** Proxy understanding^a^ of code status at the time of interview.

Proxy understanding of code status^b^	Actual code status at time of interview			
	Full code	DNR only	DNR / DNI	Total^c^
	(N = 93)	(N = 9)	(N = 9)	(N = 111)
If a life-support machine is needed they’ll use it. **OR** They’re using life-support machines to keep him/her alive. If his/her heart stops they’ll do CPR.	60	1	0	61 (55%)
If a life-support machine is needed they’ll use it. **OR** They’re using life-support machines to keep him/her alive. But if his/her heart stops they won't do CPR.	3	5	0	8 (7%)
They’re trying to help him/her get better, but they will not use life support machines and they will not do CPR if his/her heart stops.	3	1	1	5 (5%)
They’re focusing on keeping him/her as comfortable as possible, even if that means he/she dies sooner.	1	1	3	5 (5%)
I don’t know.	26	1	5	32 (29%)

^a^ 69 proxies (62%) correctly identified their loved one’s code status (unshaded), 10 proxies (9%) incorrectly identified their loved one’s code status, and 32 proxies (29%) reported that they did not know how their loved one was being treated at the time of interview. Among the 10 incorrect proxies, 9 (90%) believed their patient would receive less aggressive treatment than their actual code status indicated.

^**b**^ Proxy response to the question “*Which of the following best describes how doctors and nurses in the ICU are treating [name] right now*? *(choose 1)”* The wording of response choices depended on the response to the previous question: "Is [name] on life-support right now?"

^**c**^ This question was added to the structured interview after the first 11 proxies had been enrolled and thus was only asked of 111 of the 122 proxies enrolled in the study.

There were 54 of 122 (44%, 95% CI 35% - 54%) proxies who reported a preferred code status that was concordant with actual code status, while 31 proxies (25%, 95% CI 18% - 34%) didn’t know what their patient would prefer **(Table 3)**. Among the 35 (27%, 95% CI 21% - 38%) proxies reporting a preferred code status discordant with the patient’s actual code status, 32 (91%, 95% CI 76% - 98%) reported the patient would prefer less use of life support. Patients reporting a discordance in preferred versus actual code status were similar in age, race, years of education, and ICU day at the time of interview (all P>0.20; all Cohen’s *d* effect sizes <0.40) **(S2 Table)**. Neither limiting analyses to proxies who provided a response other than “I don’t know”, nor excluding family members who were not legal healthcare proxies substantially changed these findings **(S4 and S5 Tables)**.

**Table 3:** Concordance^a^ between preferred code status and actual code status.

Preferred code status^b^	Actual code status at time of interview			
	Full code	DNR only	DNR/DNI	Total
	(N = 103)	(N = 9)	(N = 10)	(N = 122)
Use life-support machines to keep me alive no matter what. If my heart stops, do CPR.	46	1	1	48 (39%)
Use life-support machines to keep me alive no matter what. But if my heart stops don’t do CPR.	6	1	1	8 (7%)
Try to help me get better, but don’t use life support machines and if my heart stops don’t do CPR.	14	5	3	22 (18%)
Focus on keeping me as comfortable as possible, even if that means I die sooner.	7	0	4	11 (9%)
I don’t know what he/she would say	28	2	1	31 (25%)
Declined to respond	2	0	0	2 (2%)

^a^ 54 proxies (44%—white cells) reported a concordant code status, 33 proxies (27%—blue cells) were unsure of their patient’s preferred code status or declined the question, and 35 proxies (29%—orange and green cells) reported a discordant code status. Among discordant proxies, 3 (9%) reported that the patient would prefer greater use of life support (green), 32 proxies (91%) reported the patient would prefer to forego at least one form of life support (orange).

^b^ Preferred code status was obtained via multiple-choice response to the following question: “*In your opinion*, *how does [name] want doctors and nurses in the ICU to treat him/her*? *Which of the following statements sounds most like what [name] would say*?*”*

There were 111 proxies who answered survey questions about both their loved ones preferred code status and current code status. Within the 69 proxies who correctly identified their patient’s current code status, 15 (22%) reported a discordant preferred code status. In contrast, among the 42 proxies who could not identify their patient’s code status, 18 (43%) reported a discordant preferred code status.

When asked to identify their patient’s primary goal of care from a list of goals derived from previous research categorizing goals of hospitalized patients, 106 proxies (88%) chose one of the following options: be cured, live longer, improve health, maintain health, accomplish a personal life goal **(Table 1)**. There were 13 proxies (11%) who selected “to be comfortable”, 2 proxies (2%) were unsure of their patient’s goal, and 1 (1%) refused the question. Among the 70 proxies (57%) who reported that their patient was not on life support at the time of interview, 13 (19%) were mechanically ventilated, 3 (4%) were receiving continuous renal replacement therapy, and 4 (6%) were receiving both. Among the 106 proxies who did not endorse being comfortable as the patient’s primary goal, 24 (23%) believed the patient would not want any life support machines or CPR, 8 (8%) did not want CPR, and 27 (25%) were unsure of the patient’s wishes **(Table 4)**.

**Table 4:** Proxy-reported preferred code status in patients for whom comfort was not the prioritized goal of care^a^.

	N = 106
Use life-support machines to keep me alive no matter what. If my heart stops, do CPR.	46 (43%)
Use life-support machines to keep me alive no matter what. But if my heart stops don’t do CPR.	8 (7%)
Try to help me get better, but don’t use life support machines and if my heart stops don’t do CPR.	18 (17%)^b^
Focus on keeping me as comfortable as possible, even if that means I die sooner.	6 (6%)^c^
I don’t know what he/she would say.	27 (25%)
Declined to respond	1 (1%)

**Abbreviations:** CPR, cardiopulmonary resuscitation.

^**a**^ Proxies for these patients selected the following options as best describing their patient’s goal of care: To be cured, To live longer, To improve health, To maintain health, and To accomplish a personal life goal.

^**b**^ 9 (50%) of these pts were on life support at the time of interview.

^**c**^ 2 (33%) of these pts were on life support at the time of interview.

## Discussion

In this study of 122 proxy decision-makers in a single MICU, 38% of proxies could not identify their patient’s code status when described using lay terminology, and less than half believed that the patient would want the actual code status recorded in the EMR. Among proxies reporting a discordant code status, 91% reported that the patient would prefer to forego life support permitted by the code status recorded in the EMR. Among ICU proxies, 98% reported that they knew their patient’s prioritized goal of care, but only 75% provided a response other than “I don’t know” when asked about the patient’s preferred code status. Proxies who were incorrect or unsure (vs were correct) about the patient’s actual code status were not substantially different based on readily identifiable characteristics of the proxy or patient. Patients and proxies characteristics also were not associated with concordance between preferred and actual code status.

Code status orders dictate how clinicians respond when a patient develops acute respiratory failure or experiences a cardiac arrest. By default, hospitalized patients are “full code”, meaning that they will be intubated and resuscitated, unless there is a code status order specifically instructing clinicians not to perform these interventions. Guidelines issued by ethical and professional associations recommend that code status orders be discussed when a provider anticipates that a patient may experience cardiac arrest.[21,22] Previous research suggests significant heterogeneity among providers in whether they choose to discuss code status at admission, [23–26] many proxies don’t understand what CPR entails, and nearly one third do not know how the medical team will respond to a patient with a DNR order.[27]

We are not aware of previous publications evaluating whether proxies of adult ICU patients know their code status. Proxy reports of patient’s prioritized goals of care in this study were similar to a previous study conducted in an academic MICU in Iowa, USA despite substantial differences in the demographics of these two patient populations.[18] In a recent study conducted in 12 acute care hospitals in Canada comparing preferred vs. actual code status, 30% of elderly patients and 31% of their families reported a code status that matched the actual code status in the EMR.[28] Our results were very similar this Canadian study (29% vs 31%) despite patients in our study being much younger, >50% non-white, and having much higher in-hospital mortality. In contrast, the frequency of discordant code status in our study was higher than the prior study in Iowa (29% vs 16%).[29]

Racial and socio-economic differences in end-of-life care in the ICU are well documented,[30–32] but neither race nor indicators of socio-economic status were significantly associated with code status discordance in this study. We also found that proxies with post-graduate education were no more likely to understand their patient’s code status than proxies with less formal education (62% vs 62%, P = 1.0). Hence, discussions of current patient status, prognosis, and treatment options using simple, direct language are appropriate regardless of proxy demographics. [33,34]

Proxies of critically ill patients are frequently, and quite appropriately, sad or anxious. It is sometimes assumed that these strong emotions impede their judgement or ability to understand basic information, and make them inappropriately optimistic. This is supported by a study which found that when asked about a loved one’s prognosis, 43% of ICU proxies were more optimistic than their intensivist while only 10% were more pessimistic than their intensivist.[35] But emotion seems an unlikely explanation for why among the families of full-code patients, 26% reported their loved one would prefer less use of life support, 27% said they were unsure what kind of care their loved one would choose, and 35% were unsure or assumed CPR would not be performed. These responses do not fit the prevailing image of the emotional proxy who insists their family member would want all possible interventions to extend their life.

### Origins of discordance between EMR orders and the proxy-reported preferred code status

The origins of discordance between preferred vs actual code status in this cohort are unknown. However there are several possible explanations. First, clinicians may have been unaware that some patients’ preferred code status had changed since MICU admission. Second, it is possible some patients had never been able to communicate, and their proxy had not been asked about preferred code status. This is consistent with a recent analysis of recorded meetings between intensivists and the families of critically ill patients that found patients’ prior treatment preferences were discussed in only 19% of meetings.[36] Third, presenting code status as a multiple choice question, in our study, may have normalized treatment limitations and contributed to the frequency of discordant responses. The framing of treatment options and the environmental conditions under which choices are made can have dramatic effects on patient and families choices.[37,38] Proxies may have changed their minds about what the patient would prefer after hearing all the code status options in the context of a multiple-choice question without a default response.[39]

Fourth, in some cases proxies may have been incorrect about the patient’s preference, and the actual code status in the EMR reflected a prior discussion between the patient and clinician. We did not interview patients because this study was embedded within a larger study specifically interested in medical proxies.[40] However proxies for ICU patients are very frequently asked to make decisions [9] since most ICU patients lack decisional capacity at some point during their ICU stay, and the vast majority of end-of-life decisions are made by proxies.[8,41,42] Therefore, the patient’s current code status and preferences previously expressed to ICU clinicians should be disclosed to proxies unless the patient has specifically requested otherwise.

Finally, the single proxy who reported that their patient would prefer to be full code despite a DNR/DNI order in the EMR was demonstrating strict substituted judgment for their patient. Specifically, this proxy explained that their unconscious spouse would ask for “[illicit] drugs and cigarettes,” want to be full code, and try to prematurely leave the hospital against medical advice. However the spouse’s death was imminent, and the proxy felt certain that prioritizing comfort, and foregoing intubation and resuscitation from cardiac arrest, was appropriate.

Failure to elicit patient goals and treatment preferences is potentially harmful even when patients do not require life support interventions. In a randomized survey of U.S. internal medicine residents, respondents were significantly less likely to pursue dialysis, bronchoscopy, and surgical consultation for a patient with a DNR order because of assumptions about these patients’ goals of care.[43] Conversely, clinicians may assume that patients without a DNR order are willing to accept invasive treatments in pursuit of longevity and fail to recognize that each significant preference-sensitive decision requires shared decision-making–a challenge referred to as “decision recognition”.[44] Importantly, a modified Delphi consensus process identified common preference-sensitive decisions that should trigger clinicians to clarify patient goals and consider initiating shared decision-making in the context of critical illness. [45]

### Eliciting patient goals vs code status

The U.S. National Academy of Medicine recommends that clinicians work “with patients and families to ensure that care provided matches closely with each individual’s goals.”[46] Interestingly, only 2% of proxies in this study reported they were unsure of their patient’s goal of care, but 26% were unsure whether their loved one would accept the use of life support machines and CPR to help achieve that goal. This difference illustrates proxy confidence about their patient’s values and how such confidence may not translate into decisions about specific treatments without clinician guidance.

### Implications for clinical research and health services researchers

Research evaluating whether patients are receiving care consistent with their goals and preferences is challenging.[47,48] Patient goals are often personal, difficult to categorize, dynamic, [16,17,49] and often not documented in the electronic medical record (EMR) in a form that’s easily extracted or analyzed.[50–52] Consequently, clinical researches and health services researchers frequently use code status, which is almost universally available in EMRs as a categorical variable, as an indicator of patient treatment preferences.[53,54] While code status is often the only information on patient treatment preferences systematically recorded and easily extractable from the EMR, our results suggest it is a weak and imperfect indicator of treatment preference. Therefore, prospective clinical studies that are able to interview patients and proxies should ask about goals and treatment limitations (i.e. preferred code status) directly using lay terminology.

### Implications for clinicians

The majority of proxies who reported that the patients’ most important goal was not comfort, also reported the patient would not choose to be full code or were unsure of the patient’s preferred code status. Hence, when a proxy says their patient would want to “go home”, “get better”, or “live longer” clinicians should not assume the patient would choose to be full code or would consent to invasive procedures. Rather, avoid premature closure, ask additional questions to determine whether the patient would accept the use of life support and CPR in an attempt to achieve their goals, and then enquire whether the proxy would like to know your professional recommendation. This is consistent with current recommendations to avoid evoking unrealistic aspirations with the question: ‘What would your loved one want?’ and instead focus conversations with proxies on how the patient would feel about likely clinical options and outcomes within the bounds of clinical reality.[55] Patient goals of care and treatment limitations are distinct attributes requiring separate querying and deliberation. Finally, clinicians should not assume that proxies will tell clinicians when they believe the current code status is not aligned with their patient’s preferences. We identified 15 proxies who correctly identified their patient’s current code status, reported it was not what they believed the patient would want, but did not notify the ICU clinical team. This is consistent with a recent study of current and former ICU proxies in which less than 50% reported being comfortable speaking up when they believed their love one would prefer less aggressive, or more aggressive medical care than they were currently receiving.[56]

### Implications for patients and family caregivers

Critically ill patients and their proxies can help ensure that life support interventions are used in ways that are consistent with patient preferences by actively engaging ICU clinicians. This means asking direct questions about whether patient goals are likely to be achievable, expected long-term patient outcomes, and whether physicians expect life support interventions to help achieve valued goals and outcomes. Patients and their proxies may need to initiate conversations about their goals and preferences multiple times, and should not assume that these conversations are conveyed across treatment teams or during physician hand-offs[57].

### Limitations, strengths and next steps

This study was conducted in a single MICU. Both within and between ICUs, there is great variability in how clinicians discuss,[24,58] and interpret[59–61] code status orders. As a next step, we encourage replicating this study using a multi-center design. We also could not determine the incidence or content of discussions that occurred between patients, proxies, and providers prior to enrollment because the majority of these discussions are not consistently documented, as common in many ICUs [50–52]. The study did not enroll ICU patients and thus could not determine how frequently proxies correctly identified patient goals and preferences about the use of life-support. We also did not analyze responses to open-ended questions about the patient’s goals of care or interrogate how proxies interpreted the word “life support” in survey questions. Finally, we did not ask proxies how confident they were in their answers, although the response option “I don’t know” was used to discourage guessing. Study strengths include enrolling a socio-economically diverse study population and addressing questions that are central to both clinical practice and research.

## Conclusions

In conclusion, code status orders were poor indicators of ICU patient goals of care and treatment preferences as reported by their proxy decision-makers. More than 1 in 3 healthcare proxies who were physically present in the ICU were either incorrect or unsure about their patient’s current code status. Readily identifiable characteristics, such as age, gender, race, and education level, were not associated with discordance in preferred vs actual code status, or with proxy misunderstanding or uncertainty about actual code status.

## Supporting information

S1 Fig Survey questions about patient goals and preferences. (PDF)

S1 Table Proxy and patient characteristics by understanding of code status (n = 111). (PDF)

S2 Table Proxy and patient characteristics by code status concordance (n = 122). (PDF)

S3 Table Proxy and patient characteristics by code status concordance excluding proxies who were unsure of the patient’s preferred code status (n = 89). (PDF)

S4 Table Proxy and patient characteristics by code status concordance for the subset of interviews with legal healthcare proxies (n = 79). (PDF)

S5 Table Proxy and patient characteristics by understanding of code status for the subset of interviews with legal healthcare proxies (n = 72). (PDF)
