# Supplementary material for: More than one in three proxies do not know their loved one’s current code status: An observational study in a Maryland ICU
Source: PLoS One. 2019 Jan 30;14(1):e0211531. doi: 10.1371/journal.pone.0211531 (PMC6353188; doi:10.1371/journal.pone.0211531)
Supplement: S1 Fig — (PDF) [file pone.0211531.s001.pdf]

Figure S1

PAB-Pilot Form 3: Family Interview

Subject ID - 2 ____________________

Data collector Initials ______________

Date: ___________________________

21. How do you think [patient's name] would describe his/her main goal right now?

____

____

____

22. In your opinion, which one of the following options best describes [patient's name] goal right now?

- ☐ To be cured
- ☐ To improve health
- ☐ To be comfortable
- ☐ Unsure
- ☐ To live longer
- ☐ To maintain health
- ☐ To accomplish a particular personal life goal (ex. wedding, birth)
- ☐ Refused

23. In your opinion, how does [name] want doctors and nurses in the ICU to treat him/her? Which of the following statements sounds most like what [name] would say? (choose 1)

- ☐1 Use life-support machines to keep me alive no matter what. If my heart stops, do CPR.
- ☐2 Use life-support machines to keep me alive no matter what, but if my heart stops don't do CPR.
- ☐3 Try to help me get better, but don't use life support machines and if my heart stops don't do CPR.
- ☐4 Focus on keeping me as comfortable as possible, even if that means I die sooner.
- ☐5 I don't know what he/she would say.
- ☐6 Refused

24. Is [name] on life-support right now?

☐1 Yes ☐0 No ☐2 Unsure ☐3 Refused

25. [ONLY if answer to question 24 if "Yes"]: Which of the following best describes how doctors and nurses in the ICU are treating [name] right now? (choose 1)

- ☐1 They're using life-support machines to keep him/her alive. If his/her heart stops they'll do CPR.
- ☐2 They're using life-support machines to keep him/her alive, but if his/her heart stops they won't do CPR.
- ☐3 They're focusing on keeping him/her as comfortable as possible, even if that means he/she dies sooner.
- ☐4 I don't know.
- ☐5 Refused

26. [ONLY if answer to question 24 if "No" or "Unsure"]: Which of the following best describes how doctors and nurses in the ICU are treating [name] right now? (choose 1)

- ☐1 If a life-support machine is needed they'll use it. If his/her heart stops they'll do CPR.
- ☐2 If a life-support machine is needed they'll use it. But if his/her heart stops they won't do CPR.
- ☐3 They're trying to help him/her get better, but they will not use life support machines and they will not do CPR if his/her heart stops.
- ☐4 They're focusing on keeping him/her as comfortable as possible, even if that means he/she dies sooner.
- ☐5 I don't know.
- ☐8 Refused
