# Supplementary material for: More than one in three proxies do not know their loved one’s current code status: An observational study in a Maryland ICU
Source: PLoS One. 2019 Jan 30;14(1):e0211531. doi: 10.1371/journal.pone.0211531 (PMC6353188; doi:10.1371/journal.pone.0211531)
Supplement: S1 Table — (PDF) [file pone.0211531.s002.pdf]

## Table S1: Proxy and patient characteristics by understanding of code status (n=111)[d]

| | Understanding of actual code status | | | |
|---|---|---|---|---|
| | Correct | Incorrect or unsure | P-value[a] | Effect size[a] |
| **Proxy and interview characteristics** | **(N = 69)** | **(N = 42)** | | |
| Age, median (IQR)[b] | 51 (39,61) | 53 (39,64) | 0.39 | 0.17 |
| Female, n (%)[b] | 46 (67%) | 28 (67%) | 0.88 | 0.07 |
| Years of education, median (IQR) | 14 (12,16) | 14 (12,16) | 0.83 | 0.17 |
| Self-identified race, n (%) [b] | | | | |
| Black or African American | 36 (52%) | 15 (36%) | | |
| White | 27 (39%) | 23 (55%) | 0.22 | 0.35 |
| Other | 5 (7%) | 2 (5%) | | |
| Relation to Patient, n (%)[c] | | | | |
| Spouse/Partner | 28 (41%) | 17 (40%) | | |
| Adult child | 20 (29%) | 13 (31%) | 0.80 | 0.19 |
| Parent | 7 (10%) | 6 (14%) | | |
| Other | 14 (20%) | 6 (14%) | | |
| ICU day during interview, median (IQR) | 3 (2,4) | 3 (2,4) | 0.53 | 0.17 |
| *"Have you ever supported a loved one in an ICU before?"*, n (%) [b] | | | | |
| Yes | 48 (70%) | 24 (57%) | 0.23 | 0.29 |
| **Patients characteristics & outcomes** | | | | |
| Age, median (IQR) | 58 (47,67) | 58 (50,70) | 0.46 | 0.10 |
| Female, n (%) | 31 (45%) | 20 (48%) | 0.94 | 0.05 |
| Median income of zip code in $US 1000s, median (IQR)[e] | 59 (36, 85) | 57 (36,68) | 0.60 | 0.08 |
| Location prior to hospitalization, n (%) [b] | | | | |
| Home (independent) | 47 (68%) | 25 (60%) | | |
| Home (with assistance) | 16 (23%) | 14 (33%) | 0.51 | 0.23 |
| Not home | 5 (7%) | 3 (7%) | | |
| Admission diagnosis, n (%) [b] | | | | |
| Respiratory failure | 33 (48%) | 21 (50%) | | |
| Sepsis | 9 (13%) | 11 (26%) | 0.05 | 0.98 |
| Gastrointestinal | 6 (9%) | 5 (12%) | | |
| Other | 17 (25%) | 3 (7%) | | |
| In-hospital death, n (%) | 21 (30%) | 10 (24%) | 0.59 | 0.16 |

**Abbreviation:** ICU, Intensive care unit; IQR, Interquartile Range; USD, United States Dollar

[a] Absolute effect size = absolute value of difference in means or proportions divided by standard error. P-values obtained from the Wilcoxon-Mann-Whitney two-sample test for continuous values, and the Chi-square test for categorical values with Fisher's exact test used for cell-sizes <10.

[b] Proxies declined to report age (n = 2), sex (n = 2), race (n = 4), and prior experience as an ICU proxy (n=3). Location prior to hospitalization missing for 1 patient and admission diagnosis missing for 6 patients.

[c] Percentages do not sum to 100% due to rounding.

[d] The question "*Which of the following best describes how doctors and nurses in the ICU are treating [name] right now? (choose 1)*" was added to the structured interview after the first 11 proxies had been enrolled and thus was only asked of 111 of the 122 proxies enrolled in the study.
