# Supplementary material for: More than one in three proxies do not know their loved one’s current code status: An observational study in a Maryland ICU
Source: PLoS One. 2019 Jan 30;14(1):e0211531. doi: 10.1371/journal.pone.0211531 (PMC6353188; doi:10.1371/journal.pone.0211531)
Supplement: S2 Table — (PDF) [file pone.0211531.s003.pdf]

Table S2: Proxy and patient characteristics by code status concordance (n=122)

	Preferred vs actual code status			
	Concordant or unsure	Discordant	P-value[a]	Effect size[a]
**Proxy and interview characteristics**	**(N = 87)**	**(N = 35)**		
Age, median (IQR)[b]	51 (39,62)	52 (41,58)	0.78	0.08
Female, n (%)[b]	57 (66%)	26 (74%)	0.57	0.15
Years of education, median (IQR)	14 (12,16)	14 (12,16)	0.66	0.08
Self-identified race, n (%)[b]				
Black or African American	41 (47%)	14 (40%)		
White	35 (40%)	20 (57%)	0.28	0.34
Other	7 (8%)	1 (3%)		
Relation to Patient, n (%)[c]				
Spouse/Partner	31 (36%)	15 (43%)		
Adult child	29 (33%)	11 (31%)	0.87	0.18
Parent	10 (11%)	4 (11%)		
Other	17 (20%)	5 (14%)		
ICU day during interview, median (IQR)	3 (2,4)	3 (2,4)	0.64	0.08
"Have you ever supported a loved one in an ICU before?", n (%)[b]				
Yes	52 (60%)	23 (66%)	0.65	0.12
**Patients characteristics & outcomes[d]**				
Age, median (IQR)	58 (48,69)	58 (46,72)	0.81	0.08
Female, n (%)	44 (51%)	16 (46%)	0.78	0.09
Median income of zip code in $US 1000s, median (IQR)[e]	58 (35, 74)	55 (48, 84)	0.64	0.14
Location prior to hospitalization, n (%)[b]				
Home (independent)	61 (70%)	20 (57%)		
Home (with assistance)	18 (21%)	13 (37%)	0.22	0.39
Not home	7 (8%)	2 (6%)		
Admission diagnosis, n (%)[b]				
Respiratory failure	39 (45%)	17 (49%)		
Sepsis	17 (20%)	7 (20%)	0.64	0.29
Gastrointestinal	6 (7%)	5 (14%)		
Other	19 (22%)	6 (17%)		
In-hospital death, n (%)	27 (31%)	7 (20%)	0.31	0.25

Abbreviation: ICU, Intensive care unit; IQR, Interquartile Range; USD, United States Dollar

[a] Absolute effect size = absolute value of difference in means or proportions divided by standard error. P-values obtained from the Wilcoxon-Mann-Whitney two-sample test for continuous values, and the Chi-square test for categorical values with Fisher's exact test used for cell-sizes <10.

[b] Proxies declined to report age (n = 2), sex (n = 2), race (n = 4), and prior experience as an ICU proxy (n=3). Location prior to hospitalization missing for 1 patient and admission diagnosis missing for 6 patients.

[c] Percentages do not sum to 100% due to rounding.

[d] 2 proxies were interviewed for 11 patients, creating 11 pairs of proxies independently answering questions about the same patient at different times during the ICU stay.

[e] US Census Bureau 2010-2014; $41,819 median household income for Baltimore City; $74,194 median household income for Maryland state. No zip code was provided for 1 non-American patient.
