# Supplementary material for: More than one in three proxies do not know their loved one’s current code status: An observational study in a Maryland ICU
Source: PLoS One. 2019 Jan 30;14(1):e0211531. doi: 10.1371/journal.pone.0211531 (PMC6353188; doi:10.1371/journal.pone.0211531)
Supplement: S3 Table — (PDF) [file pone.0211531.s004.pdf]

Table S3: Proxy and patient characteristics by code status concordance excluding proxies who were unsure of the patient's preferred code status (n = 89)

	Preferred vs actual code status			
	Concordant	Discordant	P-value*	Effect size*
**Proxy and interview characteristics**	**(N = 54)**	**(N = 35)**		
Age, median (IQR)†	50 (38,62)	52 (41,58)	0.77	0.08
Female, n (%)†	34 (63%)	26 (74%)	0.38	0.25
Years of education, median (IQR)	14 (12,17)	14 (12,17)	0.68	0.08
Self-identified race, n (%)†				
Black or African American	28 (52%)	14 (40%)		
White	21 (39%)	20 (57%)	0.35	0.35
Other	3 (6%)	1 (3%)		
Relation to Patient, n (%)‡				
Spouse/Partner	20 (37%)	15 (43%)		
Adult child	15 (28%)	11 (31%)	0.63	0.27
Parent	5 (9%)	4 (11%)		
Other	14 (26%)	5 (14%)		
ICU day during interview, median (IQR)	3 (2,3)	3 (2,4)	0.62	0.08
"Have you ever supported a loved one in an ICU before?", n (%)†				
Yes	33 (61%)	23 (66%)	0.87	0.09
**Patients characteristics & outcomes**				
Age, median (IQR)	58 (47, 69)	58 (46,72)	0.93	0.05
Female, n (%)	29 (54%)	16 (46%)	0.60	0.15
Median income of zip code in $US 1000s, median (IQR)§	59 (35, 85)	55 (48, 84)	0.77	0.07
Location prior to hospitalization, n (%)†				
Home (independent)	36 (67%)	20 (57%)		
Home (with assistance)	12 (22%)	13 (37%)	0.35	0.36
Not home	5 (9%)	2 (6%)		
Admission diagnosis, n (%) †				
Respiratory failure	25 (46%)	17 (49%)		
Sepsis	8 (15%)	7 (20%)	0.50	0.38
Gastrointestinal	3 (6%)	5 (14%)		
Other	13 (24%)	6 (17%)		
In-hospital death, n (%)	17 (31%)	7 (20%)	0.34	0.28

Abbreviation: ICU, Intensive care unit; IQR, Interquartile Range; USD, United States Dollar

*Absolute effect size = absolute value of difference in means or proportions divided by standard error. P-values obtained from the Wilcoxon-Mann-Whitney two-sample test for continuous values, and the Chi-square test for categorical values with Fisher's exact test used for cell-sizes <10.

† Proxies declined to report age (n = 1), race (n = 2), and prior experience as an ICU proxy (n=3). Location prior to hospitalization missing for 1 patient and admission diagnosis missing for 5 patients.

‡ Percentages do not sum to 100% due to rounding.

§ US Census Bureau 2010-2014; $41,819 median household income for Baltimore City; $74,194 median household income for Maryland state. No zip code was provided for 1 non-American patient.
