# Supplementary material for: More than one in three proxies do not know their loved one’s current code status: An observational study in a Maryland ICU
Source: PLoS One. 2019 Jan 30;14(1):e0211531. doi: 10.1371/journal.pone.0211531 (PMC6353188; doi:10.1371/journal.pone.0211531)
Supplement: S4 Table — (PDF) [file pone.0211531.s005.pdf]

Table S4: Proxy and patient characteristics by code status concordance for the subset of interviews with legal healthcare proxies (n = 79)

	Preferred vs actual code status			
	Concordant or unsure	Discordant	P-value*	Effect size*
**Proxy and interview characteristics**	**(N = 33)**	**(N = 46)**		
Age, median (IQR)†	54 (45, 62)	54 (49, 60)	0.80	0.09
Female, n (%)†	19 (58%)	33 (72%)	0.29	0.31
Years of education, median (IQR)	14 (12, 16)	14 (12, 16)	0.75	0.09
Self-identified race, n (%)†				
Black or African American	15 (45%)	16 (35%)		
White	17 (52%)	26 (56%)	0.64	0.28
Other	1 (3%)	3 (7%)		
Relation to Patient, n (%)				
Spouse/Partner	20 (61%)	25 (54%)		
Adult child	7 (21%)	12 (26%)	0.87	0.21
Parent	3 (9%)	6 (13%)		
Other	3 (9%)	3 (7%)		
ICU day during interview, median (IQR)	3 (2, 3)	3 (3, 4)	0.10	0.09
"Have you ever supported a loved one in an ICU before?", n (%)†				
Yes	22 (67%)	29 (63%)	0.78	0.12
**Patients characteristics & outcomes**				
Age, median (IQR)	59 (48, 69)	58 (49, 70)	0.97	<0.01
Female, n (%)	14 (42%)	19 (41%)	1.0	0.02
Median income of zip code in $US 1000s, median (IQR)§	59 (35, 96)	62 (49, 82)	0.69	0.05
Location prior to hospitalization, n (%)†				
Home (independent)	27 (82%)	31 (67%)		
Home (with assistance)	4 (12%)	13 (28%)	0.21	0.49
Not home	1 (3%)	2 (4%)		
Admission diagnosis, n (%) †				
Respiratory failure	12 (36%)	22 (48%)		
Sepsis	7 (21%)	10 (22%)	0.73	0.32
Gastrointestinal	2 (6%)	5 (11%)		
Other	8 (24%)	8 (17%)		
In-hospital death, n (%)	11 (33%)	12 (26%)	0.65	0.17

Abbreviation: ICU, Intensive care unit; IQR, Interquartile Range; USD, United States Dollar

*Absolute effect size = absolute value of difference in means or proportions divided by standard error. P-values obtained from the Wilcoxon-Mann-Whitney two-sample test for continuous values, and the Chi-square test for categorical values with Fisher's exact test used for cell-sizes <10.

† Proxies declined to report age (n = 1), race (n = 1), and prior experience as an ICU proxy (n=1). Location prior to hospitalization missing for 1 patient and admission diagnosis missing for 5 patients.

‡ Percentages do not sum to 100% due to rounding.

§ US Census Bureau 2010-2014; $41,819 median household income for Baltimore City; $74,194 median household income for Maryland state. No zip code was provided for 1 non-American patient.
