# Supplementary material for: More than one in three proxies do not know their loved one’s current code status: An observational study in a Maryland ICU
Source: PLoS One. 2019 Jan 30;14(1):e0211531. doi: 10.1371/journal.pone.0211531 (PMC6353188; doi:10.1371/journal.pone.0211531)
Supplement: S5 Table — (PDF) [file pone.0211531.s006.pdf]

Table S5: Proxy and patient characteristics by understanding of code status for the subset of interviews with legal healthcare proxies (n = 72)§

	Understanding of actual code status			
	Correct	Incorrect or unsure	P-value*	Effect size*
**Proxy and interview characteristics**	**(N = 46)**	**(N = 26)**		
Age, median (IQR)	54 (45, 62)	56 (49, 62)	0.54	0.13
Female, n (%)	31 (67%)	16 (62%)	0.81	0.12
Years of education, median (IQR)	14 (12, 16)	14 (12, 16)	0.87	0.13
Self-identified race, n (%)				
Black or African American	21 (46%)	8 (31%)		
White	24 (52%)	16 (62%)	0.23	0.50
Other	1 (2%)	2 (8%)		
Relation to Patient, n (%)‡				
Spouse/Partner	27 (59%)	17 (65%)		
Adult child	9 (20%)	5 (19%)	0.24	0.42
Parent	4 (9%)	4 (15%)		
Other	6 (13%)	0 (0%)		
ICU day during interview, median (IQR)	3 (2, 4)	3 (2, 4)	0.48	0.13
"Have you ever supported a loved one in an ICU before?", n (%)†				
Yes	34 (74%)	15 (58%)	0.19	0.40
**Patients characteristics & outcomes**				
Age, median (IQR)	58 (46, 68)	58 (51, 68)	0.80	0.02
Female, n (%)	16 (35%)	11 (42%)	0.70	0.15
Median income of zip code in $US 1000s, median (IQR)¦¦	60 (36, 85)	60 (48, 73)	0.92	0.03
Location prior to hospitalization, n (%) †				
Home (independent)	34 (73%)	18 (69%)		
Home (with assistance)	10 (22%)	7 (27%)	0.89	0.18
Not home	1 (2%)	1 (4%)		
Admission diagnosis, n (%) †				
Respiratory failure	19 (41%)	14 (54%)		
Sepsis	8 (17%)	6 (23%)	0.34	0.42
Gastrointestinal	4 (9%)	3 (12%)		
Other	11 (24%)	2 (8%)		
In-hospital death, n (%)	14 (30%)	7 (27%)	0.96	0.08

Abbreviation: ICU, Intensive care unit; IQR, Interquartile Range; USD, United States Dollar

* Absolute effect size = absolute value of difference in means or proportions divided by standard error. P-values obtained from the Wilcoxon-Mann-Whitney two-sample test for continuous values, and the Chi-square test for categorical values with Fisher's exact test used for cell-sizes <10.

† One proxies declined to report race prior experience as an ICU proxy. Location prior to hospitalization missing for 1 patient and admission diagnosis missing for 6 patients.

‡ Percentages do not sum to 100% due to rounding.

§ The question "*Which of the following best describes how doctors and nurses in the ICU are treating [name] right now? (choose 1)*" was added to the structured interview after the first 11 proxies had been enrolled and thus was only asked of 111 of the 122 proxies enrolled in the study. Of the 111 proxies asked this question, 72 were legal healthcare proxies.

¦¦ US Census Bureau 2010-2014; $41,819 median household income for Baltimore City; $74,194 median household income for Maryland state. No zip code was provided for 1 non-American patient.
